# Glucose 6-P Dehydrogenase Overexpression Improves Aging-Induced Endothelial Dysfunction in Aorta from Mice: Role of Arginase II

**DOI:** 10.3390/ijms24043622

**Published:** 2023-02-11

**Authors:** Eva Serna, Maria D Mauricio, Teresa San-Miguel, Sol Guerra-Ojeda, David Verdú, Alicia Valls, Coralie Arc-Chagnaud, Adrián De la Rosa, José Viña

**Affiliations:** 1 Department of Physiology, Faculty of Medicine, University of Valencia, 46010 Valencia, Spain; 2 Department of Pathology, Faculty of Medicine, University of Valencia, 46010 Valencia, Spain

**Keywords:** arginase, nitric oxide, vascular reactivity, aging, G6PD, aorta

## Abstract

The increase of vascular arginase activity during aging causes endothelial dysfunction. This enzyme competes with the endothelial nitric oxide synthase (eNOS) for L-arginine substrate. Our hypothesis is that glucose 6-P dehydrogenase (G6PD) overexpression could improve the endothelial function modulating the arginase pathway in aorta from mice. For this study, three groups of male mice were used: young wild type (WT) (6–9 months), old WT (21–22 months) and old G6PD-Tg (21–22 months) mice. Vascular reactivity results showed a reduced acetylcholine-dependent relaxation in the old WT but not old G6PD-Tg group. Endothelial dysfunction was reverted by nor-NOHA, an arginase inhibitor. Mice overexpressing G6PD underexpressed arginase II and also displayed a lower activity of this enzyme. Moreover, histological analyses demonstrated that age causes a thickness of aortic walls, but this did not occur in G6PD-Tg mice. We conclude that the overexpressing G6PD mouse is a model to improve vascular health via the arginase pathway.

## 1. Introduction

Aging is defined as a universal, progressive and irreversible time-dependent decline in the intrinsic physiological process of living organisms, leading to increased vulnerability to death [1,2,3,4,5]. It supposes a main primary risk for several pathologies such as cancer, diabetes, cardiovascular disorders and neurodegenerative diseases [6,7], but the mechanisms by which all these diseases are triggered by age remain largely unknown.

Vascular aging has been identified as a cardiovascular risk factor. Particularly, arteries suffer numerous detrimental processes, which could be considered as vascular aging hallmarks [8]. Vascular aging is a gradually developing process characterized by age-related alterations in both the structural and mechanical properties of the vascular wall, accompanied by functional vascular changes leading to the loss of arterial elasticity, reduced arterial compliance and endothelial dysfunction [9,10]. Some of the most remarkable vascular aging biological characteristics are elastin fragmentation and collagen accumulation. They cause reduced vascular compliance and increased arterial pressure [11]. Moreover, there is positive feedback between arterial stiffness and hypertension [12] that is closely related to endothelial dysfunction. Finally, vascular calcifications are observed in cells of the media or intima of the arterial wall in aging [13,14]. Although all these hallmarks are very useful for predicting patients’ biological age, they need to be combined to develop an effective biomarker [8,11].

Endothelial dysfunction is mainly due to the loss of NO bioavailability. A decrease in NO bioavailability seems to be a central mechanism for endothelial dysfunction in atherosclerosis, hypertension and hypercholesterolemia [15,16,17]. However, the mechanisms by which this happens are still unclear [18]. One possible explanation is arginase upregulation. Arginase hydrolyzes l-arginine to ornithine and urea as part of the urea cycle and has the potential to modulate l-arginine bioavailability [19]. With advancing age, the expression and activity of arginase increases in the vasculature and contributes to endothelial dysfunction [18,20,21,22,23,24,25,26,27,28].

Arginase II is predominantly expressed in kidney, prostate, blood vessels, small intestine, central nervous system, skin, lactating mammary glands and hematopoietic cells [21,29,30,31,32]. Although its function is not entirely clear, it participates in L-arginine homeostasis regulation and L-ornithine production [33]. In the vessels, L-arginine is mainly metabolized by two enzymes: arginase II and endothelial nitric oxide synthase (eNOS). The first one triggers the production of L-ornithine and urea. L-ornithine could be transformed into two different new products. In the presence of the enzyme ornithine decarboxylase (ODC), it forms polyamines, which are essential for gene regulation, cell growth and membrane transport [29,31]. Moreover, when the ornithine aminotransferase (OAT) is present, the reaction produces L-proline, an amino acid involved in collagen production [31,34]. eNOS forms L-citrulline and nitric oxide (NO) [35,36,37].

Glucose-6-phosphate dehydrogenase (G6PD) is a major producer of NADPH, which plays a significant role in the maintenance of healthy vessels, acting as a cofactor for eNOS in the metabolism of L-arginine [36,38]. G6PD deficiency promotes oxidative stress and decreases GSH levels in endothelial cells culture. This deficient G6PD activity is associated with a reduction in both NADPH stores and NO bioavailability [39]. As expected, G6PD overexpression increases eNOS activity, protecting endothelial cells against increased oxidant stress [40]. However, there is no evidence of whether the G6PD model is able to modulate vascular tone or affect the arginase pathway.

We hypothesized that an increase in G6PD activity in vivo could prevent an age-associated increase in the levels of arginase in aorta and ameliorate endothelial dysfunction.

## 2. Results

### 2.1. Vascular Reactivity Study

**Figure 1:**
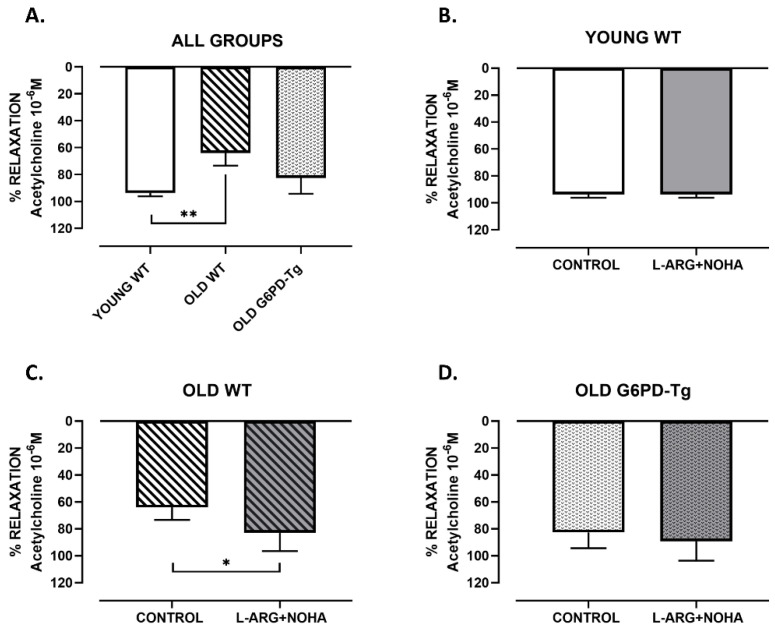
Maximal endothelium-dependent vasorelaxation in response to acetylcholine for all groups (Panel **A**) and preincubated with L-arginine 10^−3^ M and nor-NOHA 10^−5^ M in young WT, old WT and old G6PD-Tg (Panel **B**–**D**) in aortic rings. Results are expressed as % relaxation of acetylcholine 10^−6^ M and are the mean ± SD (*n* = 5–10 per group). ** *p* < 0.01 and * *p* < 0.05.

Acetylcholine (10^−6^ M) caused an endothelium-dependent relaxation in aorta from all groups (Figure 1A). Maximal vasodilation was 94 ± 2% for young wild type (WT) mice and 64 ± 9% (*p* < 0.01) for old WT mice, indicating an endothelial dysfunction with aging. However, the vasodilation for old G6PD-Tg mice was 84 ± 12%, indicating an improvement in the endothelial vasodilation (Figure 1A).

After incubation with L-arginine (10^−3^ M) plus nor-NOHA (10^−5^ M) (Figure 1), an arginase inhibitor, the relaxation induced by acetylcholine was increased in old WT mice (vasodilation of 64 ± 9% to 83 ± 14%, *p* < 0.05) (Figure 1C). In the rest of the groups, it did not show any effect (vasodilation of young WT mice from 94 ± 2% to 94 ± 2% and old G6PD-Tg mice from 83 ± 12% to 89 ± 14%) (Figure 1B,D).

These results indicate that arginase participates in endothelial dysfunction in aorta from old wild type mice but not in old G6PD-Tg mice.

### 2.2. Gene Expression Analysis

**Figure 2:**
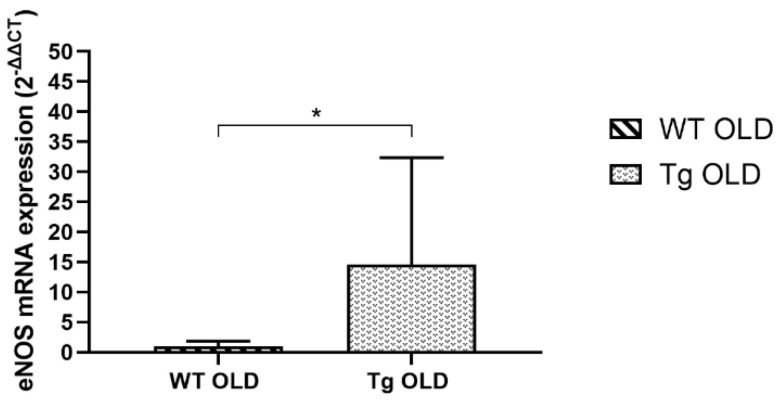
eNOS gene expression in aortic rings from old WT and old Tg mice groups studied. Results are expressed as fold change (2^−ΔΔCt^) and are the geometric mean with 95% CI (*n* = 4 per group). * *p* < 0.05.

mRNA expression of eNOS in aortic rings from old G6PD-Tg mice was 14-fold higher than in old WT mice (Figure 2).

**Figure 3:**
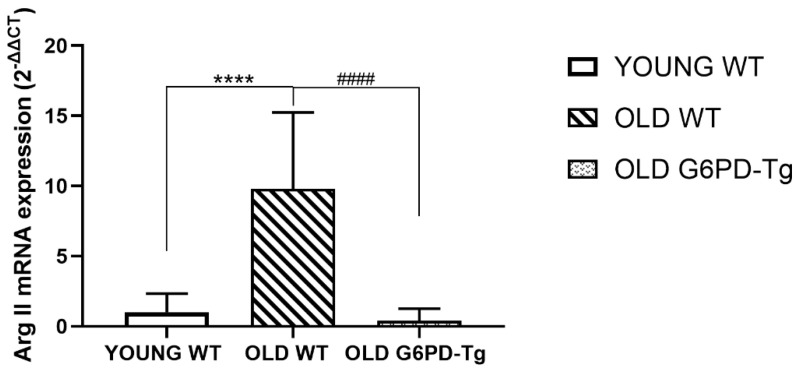
Arginase II gene expression in aortic rings for all groups studied. Results are expressed as fold change (2^−ΔΔCt^) and are the geometric mean with 95% CI (*n* = 4 per group). **** *p* < 0.0001 between WT groups. ^####^ *p* < 0.0001 between old groups.

Arginase II gene expression was increased tenfold in old WT mice compared with young WT mice. This increase was not observed in old G6PD-Tg mice (Figure 3).

### 2.3. Arginase Activity

Arginase activity from young WT, old WT and old G6PD-Tg mice was measured in aortic rings.

**Figure 4:**
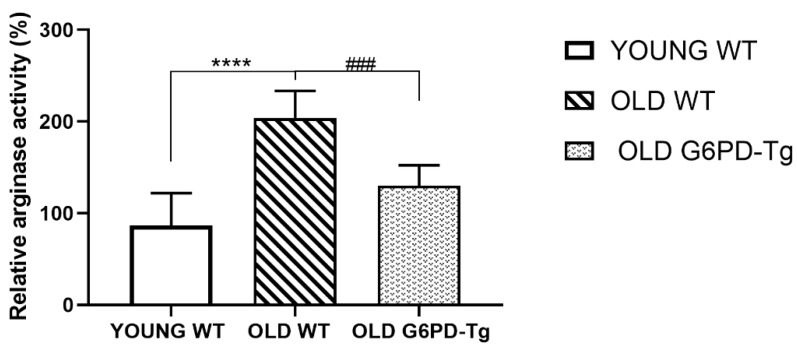
Relative arginase activity in the aortic rings. Results are means ± SD (*n* = 5–8 per group). **** *p* < 0.0001 between WT groups; ^###^ *p* < 0.001 between old groups.

Activity in old WT mice was two-fold higher than in young ones. This did not happen in G6PD-Tg mice (Figure 4). 

### 2.4. Histological Study: Arterial Wall Thickness and Collagen Deposition

**Figure 5:**
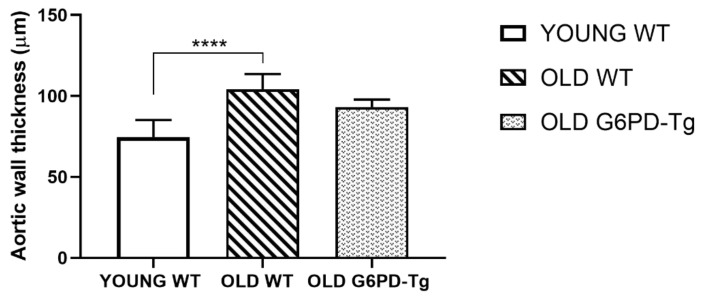
Aortic wall thickness for all groups studied. Results are means ± SD (*n* = 8–10 per group). **** *p* < 0.0001.

**Figure 6:**
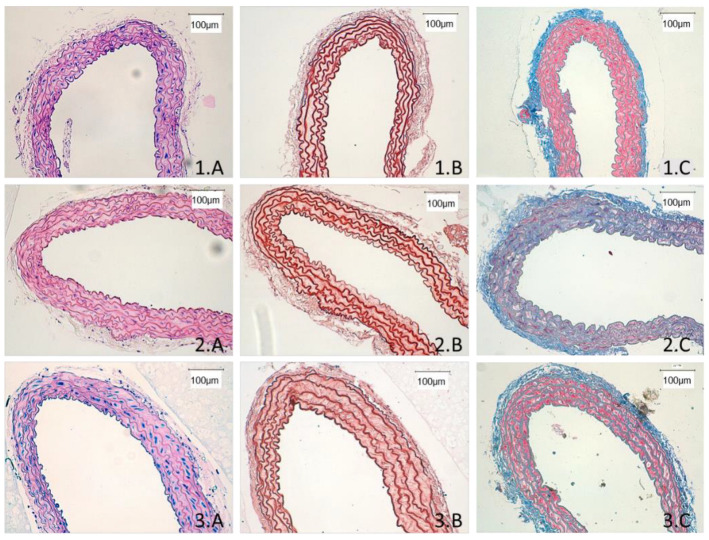
Representative microphotographs from histological sections of aorta. (**A**) Hematoxylin-eosin. (**B**) Orcein staining. (**C**) Masson’s trichrome. 1: Young WT samples. 2: Old WT samples. 3: Old G6PD-Tg samples. Results are representative of *n* = 6 per group.

Histological procedures were performed to assess changes in aortic wall thickness and structure. First, haematoxylin-eosin stained slides were used to measure the average aortic wall thickness of samples in all groups (Figure 5 and Figure 6(1.A,2.A,3.A)). Old WT mice showed 25% thicker aortic rings than young WT ones. This was partially prevented in old G6PD-Tg mice. The examination of orcein staining showed no differences in the amount, shape or disposition of elastic fibres, suggesting a similar structural organisation in all the groups (Figure 6 (1.B,2.B,3.B)).

Collagen deposition as measured by the Masson trichrome stain showed a statistically significant increase in the amount of collagen in the aorta with aging. The mean values of the staining showed that the old WT mice had 7% higher collagen levels than the young WT mice (*p* < 0.05) and 9% higher than the old G6PD-Tg group (*p* < 0.01). Those values were estimated by the histograms generated by aniline blue using FIJI software (Table 1). In addition, a qualitative observation of the samples evidenced that in both young WT and old G6PD-Tg groups, collagen was better defined and localized with a better organized disposition than in old WT samples (Figure 6(1.C,2.C,3.C)).

## 3. Discussion

The main finding of the present work is that an overexpression of the enzyme G6PD modulates arginase in the aorta from old mice, reversing the endothelial dysfunction observed with aging [41]. This dysfunction is mainly associated with a decrease in nitric oxide bioavailability [42,43], which, among other factors, is due to increased levels of asymmetric dimethylarginine (ADMA), an endogenous inhibitor of eNOS associated with frailty [44].

In aging, arginase upregulation contributes to endothelial dysfunction [20,45]. Moreover, arginase inhibitors restore NO-mediated vasodilation in rats [46] and in elderly subjects [24]. In agreement with this, our results show that the administration of L-arginine plus nor-NOHA increases the response to acetylcholine in the aorta from old WT mice. However, L-arginine plus nor-NOHA does not enhance vasodilation in response to acetylcholine in young WT or old G6PD-Tg mice. These results indicate that endothelial dysfunction in the old WT group is due to reduced L-arginine availability, secondary to increased arginase activity, and that an overexpression of the enzyme G6PD down-regulates the arginase pathway, preserving endothelial function. This was supported by the observation of an increase of arginase II gene expression and arginase activity in the aortas from old WT mice, but not in those from old G6PD-Tg mice. Furthermore, eNOS gene expression is increased in the aorta from old G6PD-Tg mice compared with old WT mice. Yepuri et al. described that there is a positive crosstalk between arginase II and p70 ribosomal protein S6 kinase 1 (S6K1), and it is involved in endothelial dysfunction in aging [45]. The effects of arginase II downregulation in our overexpressed G6PD model could contribute to a decrease in S6K1 in these animals and hence decelerate vascular aging.

Our results also support the influence of the arginase pathway in some age-related histological characteristics. The histological analysis revealed an increase in the aortic wall thickness from old WT mice, supporting the age-related increase in arginase. In contrast, the aorta from old G6PD-Tg mice showed no change in the wall thickness compared with the young WT group. Interestingly, a higher and more diffuse amount of collagen was also found in aortas from old WT mice, as expected [47], whereas the G6PD-Tg mice showed lower values, similar to those of young WT mice. Since collagen contributes to increased vascular stiffness with aging, this finding strongly suggests that G6PD overexpression has a preventive effect in this aging-related morphological feature. Our findings are carried out in mouse aorta, but isolation of primary cells would be needed to confirm whether the overexpression of G6PD is in endothelial cells, smooth muscle cells or both. However, the global effect is demonstrated in this work.

The overexpression of G6PD has been reported to be protective in the development of some age-associated conditions in mice, such as insulin resistance, neuromuscular impairment, lower DNA oxidation levels and frailty [48,49]. G6PD is the first and rate-limiting enzyme of the pentose phosphate pathway. It catalyzes the synthesis of riboses and serves as a major source of cellular NADPH. Thus, we confirm that NADPH plays an important role in the maintenance of vascular homeostasis, acting as a cofactor for eNOS in L-arginine metabolism [36,38]. 

In this work, we conclude that G6PD-Tg mice show protection against aging-associated vascular dysfunction by modulating eNOS as well as the arginase pathway.

## 4. Materials and Methods

### 4.1. Animal Model and Study Design

All experiments involving animals were reviewed and approved by the University of Valencia Ethics Committee for Research and Animal Care. Male mice were housed in groups of 3–4 in a plastic cage, under standard laboratory room conditions (temperature: 24± °C, 12/12-h light/dark cycle) with food and water ad libitum. 

G6PD transgenic mice were generated at the Spanish National Cancer Research Centre (CNIO) at the Transgenic Mice core facility and were housed at the specific pathogen free barrier areas at the CNIO Animal House core facility and the University of Valencia. 

G6PD-Tg mouse line was generated using a 20,105 Kb human genomic DNA construct containing the whole G6PD gene, including 2.5 Kb of upstream flanking sequence and 2.0 Kb of downstream flanking sequence [50]. For transgenesis, the G6PD sequence was isolated from the pBluescript vector by NotI digestion and a 0.5 to 1 ng/µL DNA solution was injected into the pronuclei of F1 hybrids (C57BL/6J × CBA) fertilized oocytes using standard microinjection procedures. The resulting offspring was analyzed for the presence of the transgene by polymerase chain reaction (PCR) using primers specific for the human G6PD gene (Forward: 5′-AAGAAGCAGACTGGAGGAGAAG-3′ and Reverse: 5′-CAGGTTGTCACTCTCAGAACAGA-3′) and that do not hybridize to the homologous mouse G6PD gene. 

One founder capable of transmitting the transgene to the progeny and that overexpressed G6PD was identified (+/+; tg), abbreviated here as G6PD-Tg. The founder G6PD-Tg mouse was backcrossed for three generations with pure C57BL6 mice; in this manner, all of the mice used in this study share a genetic background that is 93.75% C57BL6. All the C57BL6 mice were purchased from Harlan Laboratories and correspond to the sub-strain C57BL6/J-OlaHsd. After the generation of the transgenic mice in the CNIO, some animals were sent to the Physiology Department, Faculty of Medicine at the University of Valencia (Valencia, Spain) to extend the colony and perform experiments.

All research centers established the same housing conditions for avoiding variability factors related to animal care. An adaptation period of 2–3 weeks was allowed before the initiation of any of the following experimental protocols.

Cohorts of wild type (WT) and G6PD-Tg (Tg) mice were used: young WT group mice (6–9 months), old WT group mice (21–22 months) and old G6PD-Tg group mice (21–22 months).

### 4.2. Vascular Reactivity Studies

Thoracic aorta rings from G6PD-Tg and WT mice were isolated from young and old groups. The artery was cleaned with saline (0.9% NaCl) prepared with sterile water treated with diethylpyrocarbonate (DEPC), under an illuminated dissecting loupe (Wild M3C, Leica Microsystems Inc. Buffalo Grove, IL USA) in cold light.

The rings were mounted in an organ bath system to measure the isometric tension and filled with 5 mL of modified Krebs-Henseleit physiological solution composed of (in mM): NaCl 115; KCl 4.6; MgCl_2_, 6H_2_O 1,2; CaCl_2_ 2.5; NaHCO_3_ 25; glucose 11.1 and EDTA disodium 0.01. This solution was equilibrated with a gaseous mixture (95% O_2_ and 5% CO_2_) that provides a pH of 7.3–7.4. The temperature of the solution was maintained at 37 °C. The optimal passive tension used was 1 g to perform the vascular studies. Vasodilation to acetylcholine (10^−6^ M) [51] was studied in aortic rings previously contracted with noradrenaline (3 × 10^−7^ M–3 × 10^−6^ M), in the absence and presence of L-arginine (10^−3^ M) [52] plus nor-NOHA acetate (10^−5^ M), an arginase inhibitor for 30 min [20,53]. A high concentration of acetylcholine (10^−6^ M) is used to stimulate maximal NO release from endothelial cells. A decreased relaxation to this dose means maximal capacity of NO production.

### 4.3. Total RNA Extraction and Real Time Polymerase Chain Reaction (RT-PCR) Studies

Aortic segments were extracted using 300 μL of TRIzol reagent. Integrity quality was performed by Nanodrop 2000 (Agilent Technologies, Santa Clara, CA, USA), and the purity was evaluated with the 260/280 ratio. RT-PCR was performed on Taqman probes (Applied Biosystems, Foster City, CA, USA) using QuantStudio v5 (Applied Biosystems, Foster City, CA, USA), establishing the proper conditions. The housekeeping gene was GAPDH (4352338E, Applied Biosystems) and the genes studied were ARG2 (Mm00477592_m1) and eNOS (Mm00435217_m1). For the results analysis, 2^-∆∆Ct^ [54] was used.

### 4.4. Arginase Activity Studies

Arginase activity was determined in aortic tissue homogenates using the Arginase Activity Assay Kit (MAK112-1KT, Sigma-Aldrich).

Aorta segments isolated in PBS solution during the sacrifice were cleaned with saline (0.9% NaCl) prepared using sterile water with DEPC, under an illuminated dissecting loupe (Wild M3C, Leica) in cold light. This procedure was followed by the lysis of the cells with a prepared mixture (containing 100 µL of Tris-HCL 10 mM (pH 7.4), 1 µL of protease inhibitor (pepstatin and leupeptin) and 1 µL Triton X-100 0.4% *w/v*), and its homogenization using autoclaved tubes and a T 10 basic Ultra-Turrax (IKA Processing Equipment).

The quantification of arginase activity was based on the reaction of the enzyme present in the samples and the substrate buffer (8 µL of arginine buffer and 2 µL of Mn solution), to form urea and ornithine as products, after two hours of incubation at 37 °C. The urea reagent (100 µL of reagent A and 100 µL of reagent B) was added to the obtained urea, giving a colored compound, followed by a room temperature incubation of one hour, stopping the reaction. A control (blank) reaction was run in parallel, containing the same amount of sample, urea reagent and substrate buffer. The amount of colored compound was read at 430 nm in a spectrophotometer (Spectramax plus 384, Molecular Devices, San Jose, CA, USA). The data obtained was exported into a computer program (Softmax pro 6.2.2, Molecular Devices) connected to the device, and then copied into an Excel document.

### 4.5. Histological Study

Fresh aorta segments were collected, fixed in neutral-buffered formalin for 24 h and embedded in paraffin following the standard protocol of dehydration, clearing and paraf-fin infiltration in a Leica ASP300 tissue processor (Leica microsystems S.L.U., Barcelona, Spain). Paraffin blocks were built and sectioned with a microtome (Leica microsystems). Different sections were stained with hematoxylin-eosin, Artisan-Masson trichrome stain kit and Artisan-Orcein stain kit (Agilent technologies Spain, Madrid, Spain). Microphoto-graphs from the tissue samples were taken in a DMD108 photomicroscope (Leica mi-crosystems). The thickness of each aorta segment was analyzed using FIJI software (Im-ageJ 2.0); briefly, the occupied area from the most external and internal part of each aorta segment was measured. Those areas were approached as circles and the average thick-ness was estimated as the difference of the radius from both in each sample (based on [55]). Every microphotography from Masson trichrome-stained slides was processed with FIJI by setting the color deconvolution option for Masson trichrome. The background was eliminated and histograms were analyzed on channel 1 (aniline blue for collagen). 

### 4.6. Statistics

All statistics were performed using the GraphPad Prism 8 (GraphPad software, San Diego, California, USA). 

First of all, the Shapiro–Wilk test was carried out in order to asses a normal or non-normal distribution of the samples. In case of non-normal distribution, the Kruskal–Wallis non-parametric test was used to search for statistical differences between all groups, and then the Mann–Whitney test determined significances among each pair of groups in the study. When samples showed normality, one-way ANOVA was performed to find significant differences between the groups, followed by Levene’s test for equality of variances. If the test reported distinct variances, Welch’s test was used to find significances among paired groups. If there is equality of variances, statistical differences are proved with two-tailed Student’s *t*-test.

For all data, a *p* < 0.05 was accepted as significant.

## Figures and Tables

**Table 1:** Estimation of collagen by Masson’s trichrome stain.

YWT	OWT	OTg
207.9	204.7	204.6
208.5	226.6	202.9
211.5	237.7	205.2
206.9	243.2	204.4
207.0	218.6	204.6
209.8	212.6	208.2
208.6 ± 1.8	223.9 ± 14.8	205.0 ± 1.7

Mean = sum pixels/area in pixels. YWT: young WT group; OWT: old WT group; OTg: old G6PD-Tg group.
